# 2-(4-Bromo­phen­yl)-2-oxoethyl 2-meth­oxy­benzoate

**DOI:** 10.1107/S1600536811023002

**Published:** 2011-06-18

**Authors:** Hoong-Kun Fun, Ching Kheng Quah, B. Garudachari, Arun M. Isloor, M. N. Satyanarayan

**Affiliations:** a X-ray Crystallography Unit, School of Physics, Universiti Sains Malaysia, 11800 USM, Penang, Malaysia; b Organic Electronics Division, Department of Chemistry, National Institute of Technology-Karnataka, Surathkal, Mangalore 575 025, India; c Department of Physics, National Institute of Technology-Karnataka, Surathkal, Mangalore 575 025, India

## Abstract

In the title mol­ecule, C_16_H_13_BrO_4_, the dihedral angle between the benzene rings is 85.92 (10)°. In the crystal, mol­ecules are linked into chains along [100] *via* weak inter­molecular C—H⋯O hydrogen bonds.

## Related literature

For general background to and applications of phenacyl benzoate derivatives, see: Rather & Reid (1919 ▶); Sheehan & Umezaw (1973 ▶); Ruzicka *et al.* (2002 ▶); Litera *et al.* (2006 ▶); Huang *et al.* (1996 ▶); Gandhi *et al.* (1995 ▶). For standard bond-length data, see: Allen *et al.* (1987 ▶).
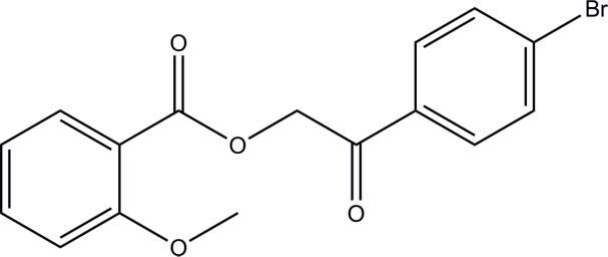


## Experimental

### 

#### Crystal data

C_16_H_13_BrO_4_
*M* *_r_* = 349.17
Orthorhombic, 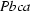

*a* = 7.8424 (5) Å
*b* = 14.6799 (9) Å
*c* = 25.7677 (14) Å
*V* = 2966.5 (3) Å^3^
*Z* = 8
Mo *K*α radiation
μ = 2.78 mm^−1^
*T* = 296 K
0.56 × 0.25 × 0.12 mm

#### Data collection

Bruker SMART APEXII DUO CCD area-detector diffractometer
Absorption correction: multi-scan (*SADABS*; Bruker, 2009 ▶) *T*_min_ = 0.306, *T*_max_ = 0.733
19358 measured reflections
4731 independent reflections
2916 reflections with *I* > 2σ(*I*)
*R*_int_ = 0.035

#### Refinement

*R*[*F* ^2^ > 2σ(*F* ^2^)] = 0.037
*wR*(*F* ^2^) = 0.095
*S* = 1.00
4731 reflections
190 parameters
H-atom parameters constrained
Δρ_max_ = 0.51 e Å^−3^
Δρ_min_ = −0.54 e Å^−3^

### 

Data collection: *APEX2* (Bruker, 2009 ▶); cell refinement: *SAINT* (Bruker, 2009 ▶); data reduction: *SAINT*; program(s) used to solve structure: *SHELXTL* (Sheldrick, 2008 ▶); program(s) used to refine structure: *SHELXTL*; molecular graphics: *SHELXTL*; software used to prepare material for publication: *SHELXTL* and *PLATON* (Spek, 2009 ▶).

## Supplementary Material

Crystal structure: contains datablock(s) global, I. DOI: 10.1107/S1600536811023002/lh5263sup1.cif

Structure factors: contains datablock(s) I. DOI: 10.1107/S1600536811023002/lh5263Isup2.hkl

Supplementary material file. DOI: 10.1107/S1600536811023002/lh5263Isup3.cml

Additional supplementary materials:  crystallographic information; 3D view; checkCIF report

## Figures and Tables

**Table 1:** Hydrogen-bond geometry (Å, °)

*D*—H⋯*A*	*D*—H	H⋯*A*	*D*⋯*A*	*D*—H⋯*A*
C2—H2*A*⋯O1^i^	0.93	2.45	3.360 (3)	165

Symmetry code: (i) 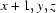.

**Fig. 1.**
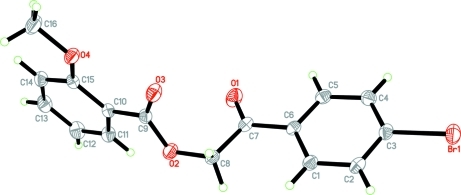
The molecular structure of the title compound showing 20% probability displacement ellipsoids for non-H atoms.

**Fig. 2.**
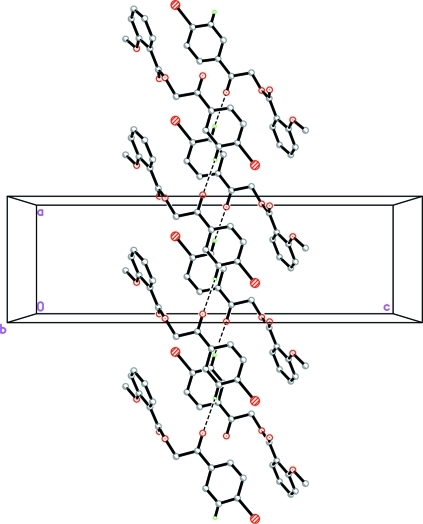
Part of the crystal structure of the title compound, viewed along the b axis. H atoms not involved in hydrogen bonds (dashed lines) have been omitted for clarity.

## supplementary crystallographic information

### Comment

Phenacyl benzoates derivatives are very important in the identification of organic acids (Rather & Reid, 1919). They undergo photolysis in neutral and mild conditions (Sheehan & Umezaw, 1973; Ruzicka *et al.*, 2002; Litera *et al.*, 2006). They find applications in the field of synthetic chemistry for the synthesis of oxazoles, imidazoles (Huang *et al.*, 1996), benzoxazepine (Gandhi *et al.*, 1995). We report herein the crystal structure of 2-(4-bromophenyl)-2-oxoethyl 2-methoxybenzoate which is potentially of commercial importance.

The molecular structure of the title compound is shown in Fig. 1. The benzene rings (C1-C6 and C10-C15) form a dihedral angle of 85.92 (10) ° . Bond lengths (Allen *et al.*, 1987) and angles are within normal ranges. In the crystal (Fig. 2), the molecules are linked into one-dimensional chains along [100] *via* weak intermolecular C2–H2A···O1^i^ hydrogen bonds (Table 1).

### Experimental

A mixture of 2-methoxybenzoic acid (1.00 g, 0.0065 mol) potassium carbonate (0.98 g, 0.0071 mol) and 2-bromo-1-(4-bromophenyl)ethanone (1.80 g, 0.0065 mol) in dimethylformamide (10 ml) was stirred at room temperature for 2 h. On cooling, colorless needle shaped crystals of 2-(4-bromophenyl)-2-oxoethyl 2-methoxybenzoate began to separate. These were collected by filtration and recrystallized from ethanol. Yield: 2.15 g, 93.8 %, *m.p.* : 388-389 K.

### Refinement

All H atoms were positioned geometrically and refined using a riding model with C—H = 0.93 – 0.96 Å and U_iso_(H) = 1.2 or 1.5 U_eq_(C). The highest residual electron density peak is located at 0.79 Å and the deepest hole is located at 0.78 Å from Br1, respectively.

### Crystal data

**Table:**

C_16_H_13_BrO_4_	*F*(000) = 1408
*M**_r_* = 349.17	*D*_x_ = 1.564 Mg m^−^^3^
Orthorhombic, *P**b**c**a*	Mo *K*α radiation, λ = 0.71073 Å
Hall symbol: -P 2ac 2ab	Cell parameters from 3938 reflections
*a* = 7.8424 (5) Å	θ = 2.8–26.5°
*b* = 14.6799 (9) Å	µ = 2.78 mm^−^^1^
*c* = 25.7677 (14) Å	*T* = 296 K
*V* = 2966.5 (3) Å^3^	Needle, colourless
*Z* = 8	0.56 × 0.25 × 0.12 mm

### Data collection

**Table:**

Bruker SMART APEXII DUO CCD area-detector diffractometer	4731 independent reflections
Radiation source: fine-focus sealed tube	2916 reflections with *I* > 2σ(*I*)
graphite	*R*_int_ = 0.035
φ and ω scans	θ_max_ = 31.0°, θ_min_ = 2.8°
Absorption correction: multi-scan (*SADABS*; Bruker, 2009)	*h* = −11→11
*T*_min_ = 0.306, *T*_max_ = 0.733	*k* = −21→14
19358 measured reflections	*l* = −37→37

### Refinement

**Table:**

Refinement on *F*^2^	Primary atom site location: structure-invariant direct methods
Least-squares matrix: full	Secondary atom site location: difference Fourier map
*R*[*F*^2^ > 2σ(*F*^2^)] = 0.037	Hydrogen site location: inferred from neighbouring sites
*wR*(*F*^2^) = 0.095	H-atom parameters constrained
*S* = 1.00	*w* = 1/[σ^2^(*F*_o_^2^) + (0.0329*P*)^2^ + 1.2401*P*] where *P* = (*F*_o_^2^ + 2*F*_c_^2^)/3
4731 reflections	(Δ/σ)_max_ = 0.001
190 parameters	Δρ_max_ = 0.51 e Å^−^^3^
0 restraints	Δρ_min_ = −0.54 e Å^−^^3^

### Special details

**Table:**

Geometry. All esds (except the esd in the dihedral angle between two l.s. planes) are estimated using the full covariance matrix. The cell esds are taken into account individually in the estimation of esds in distances, angles and torsion angles; correlations between esds in cell parameters are only used when they are defined by crystal symmetry. An approximate (isotropic) treatment of cell esds is used for estimating esds involving l.s. planes.
Refinement. Refinement of F^2^ against ALL reflections. The weighted R-factor wR and goodness of fit S are based on F^2^, conventional R-factors R are based on F, with F set to zero for negative F^2^. The threshold expression of F^2^ > 2sigma(F^2^) is used only for calculating R-factors(gt) etc. and is not relevant to the choice of reflections for refinement. R-factors based on F^2^ are statistically about twice as large as those based on F, and R- factors based on ALL data will be even larger.

### Fractional atomic coordinates and isotropic or equivalent isotropic displacement parameters (Å^2^)

**Table:**

	*x*	*y*	*z*	*U*_iso_*/*U*_eq_	
Br1	1.19995 (4)	0.063553 (17)	0.603716 (11)	0.07024 (11)	
O1	0.4583 (2)	0.13020 (14)	0.46925 (7)	0.0717 (5)	
O2	0.4899 (2)	0.19606 (9)	0.37415 (6)	0.0579 (4)	
O3	0.4628 (2)	0.04790 (10)	0.35626 (6)	0.0604 (4)	
O4	0.19571 (19)	0.00663 (10)	0.29651 (6)	0.0544 (4)	
C1	0.9183 (3)	0.14467 (14)	0.47702 (8)	0.0488 (5)	
H1A	0.9406	0.1695	0.4445	0.059*	
C2	1.0517 (3)	0.12695 (15)	0.51036 (8)	0.0517 (5)	
H2A	1.1635	0.1396	0.5007	0.062*	
C3	1.0158 (3)	0.09006 (13)	0.55825 (8)	0.0487 (5)	
C4	0.8516 (3)	0.07055 (14)	0.57344 (8)	0.0538 (5)	
H4A	0.8302	0.0457	0.6060	0.065*	
C5	0.7199 (3)	0.08825 (15)	0.53991 (8)	0.0504 (5)	
H5A	0.6087	0.0749	0.5498	0.060*	
C6	0.7504 (3)	0.12584 (13)	0.49133 (7)	0.0428 (4)	
C7	0.6034 (3)	0.14284 (14)	0.45588 (8)	0.0470 (4)	
C8	0.6423 (3)	0.17663 (14)	0.40202 (8)	0.0496 (5)	
H8A	0.7113	0.2313	0.4042	0.059*	
H8B	0.7073	0.1308	0.3835	0.059*	
C9	0.4043 (3)	0.12319 (13)	0.35557 (7)	0.0444 (4)	
C10	0.2356 (3)	0.15108 (13)	0.33476 (7)	0.0420 (4)	
C11	0.1753 (3)	0.23898 (15)	0.34381 (8)	0.0540 (5)	
H11A	0.2411	0.2790	0.3633	0.065*	
C12	0.0207 (3)	0.26793 (17)	0.32457 (9)	0.0654 (6)	
H12A	−0.0176	0.3268	0.3311	0.078*	
C13	−0.0762 (3)	0.20873 (18)	0.29561 (9)	0.0643 (6)	
H13A	−0.1803	0.2280	0.2823	0.077*	
C14	−0.0216 (3)	0.12187 (16)	0.28619 (8)	0.0551 (5)	
H14A	−0.0894	0.0827	0.2668	0.066*	
C15	0.1337 (3)	0.09149 (13)	0.30522 (7)	0.0442 (4)	
C16	0.0967 (4)	−0.05475 (17)	0.26574 (11)	0.0735 (7)	
H16A	0.1556	−0.1118	0.2627	0.110*	
H16B	0.0799	−0.0292	0.2318	0.110*	
H16C	−0.0120	−0.0644	0.2820	0.110*	

### Atomic displacement parameters (Å^2^)

**Table:**

	*U*^11^	*U*^22^	*U*^33^	*U*^12^	*U*^13^	*U*^23^
Br1	0.07526 (19)	0.05554 (15)	0.07991 (18)	0.00639 (12)	−0.03345 (13)	−0.00558 (11)
O1	0.0385 (8)	0.1055 (14)	0.0713 (10)	−0.0059 (9)	0.0015 (8)	−0.0039 (10)
O2	0.0574 (9)	0.0434 (8)	0.0729 (9)	−0.0015 (7)	−0.0214 (8)	0.0012 (7)
O3	0.0584 (9)	0.0481 (8)	0.0746 (10)	0.0109 (7)	−0.0218 (8)	−0.0110 (7)
O4	0.0577 (9)	0.0459 (7)	0.0595 (8)	−0.0012 (7)	−0.0141 (7)	−0.0071 (6)
C1	0.0433 (11)	0.0560 (11)	0.0472 (10)	−0.0059 (10)	0.0009 (9)	−0.0014 (9)
C2	0.0372 (10)	0.0562 (12)	0.0617 (12)	−0.0049 (9)	−0.0018 (9)	−0.0084 (10)
C3	0.0535 (12)	0.0381 (9)	0.0544 (11)	0.0025 (9)	−0.0136 (10)	−0.0098 (8)
C4	0.0634 (14)	0.0506 (11)	0.0474 (10)	−0.0032 (11)	−0.0006 (10)	−0.0014 (9)
C5	0.0430 (11)	0.0542 (11)	0.0540 (11)	−0.0053 (10)	0.0062 (9)	−0.0046 (9)
C6	0.0381 (9)	0.0421 (10)	0.0481 (10)	−0.0023 (8)	0.0013 (8)	−0.0093 (8)
C7	0.0398 (11)	0.0466 (10)	0.0547 (11)	−0.0035 (9)	−0.0017 (9)	−0.0102 (8)
C8	0.0444 (11)	0.0434 (10)	0.0608 (12)	−0.0064 (9)	−0.0111 (9)	0.0010 (9)
C9	0.0505 (12)	0.0430 (10)	0.0398 (9)	0.0012 (9)	−0.0048 (8)	−0.0011 (8)
C10	0.0453 (10)	0.0437 (9)	0.0371 (8)	0.0042 (8)	−0.0024 (8)	0.0010 (7)
C11	0.0603 (14)	0.0510 (11)	0.0508 (11)	0.0078 (10)	−0.0030 (10)	−0.0057 (9)
C12	0.0679 (15)	0.0613 (14)	0.0671 (14)	0.0228 (13)	−0.0041 (12)	−0.0009 (11)
C13	0.0505 (13)	0.0780 (16)	0.0646 (13)	0.0163 (12)	−0.0071 (11)	0.0099 (12)
C14	0.0486 (12)	0.0654 (14)	0.0513 (11)	−0.0005 (11)	−0.0093 (10)	0.0052 (10)
C15	0.0475 (11)	0.0490 (10)	0.0362 (8)	0.0017 (9)	0.0003 (8)	0.0040 (7)
C16	0.0819 (18)	0.0597 (14)	0.0788 (16)	−0.0071 (13)	−0.0264 (15)	−0.0160 (11)

### Geometric parameters (Å, °)

**Table:**

Br1—C3	1.900 (2)	C7—C8	1.505 (3)
O1—C7	1.203 (3)	C8—H8A	0.9700
O2—C9	1.351 (2)	C8—H8B	0.9700
O2—C8	1.423 (2)	C9—C10	1.485 (3)
O3—C9	1.197 (2)	C10—C11	1.394 (3)
O4—C15	1.356 (2)	C10—C15	1.408 (3)
O4—C16	1.430 (3)	C11—C12	1.377 (3)
C1—C2	1.378 (3)	C11—H11A	0.9300
C1—C6	1.395 (3)	C12—C13	1.375 (3)
C1—H1A	0.9300	C12—H12A	0.9300
C2—C3	1.377 (3)	C13—C14	1.367 (3)
C2—H2A	0.9300	C13—H13A	0.9300
C3—C4	1.376 (3)	C14—C15	1.386 (3)
C4—C5	1.371 (3)	C14—H14A	0.9300
C4—H4A	0.9300	C16—H16A	0.9600
C5—C6	1.389 (3)	C16—H16B	0.9600
C5—H5A	0.9300	C16—H16C	0.9600
C6—C7	1.492 (3)		
			
C9—O2—C8	115.94 (15)	H8A—C8—H8B	108.0
C15—O4—C16	118.42 (17)	O3—C9—O2	122.39 (19)
C2—C1—C6	120.95 (19)	O3—C9—C10	126.97 (18)
C2—C1—H1A	119.5	O2—C9—C10	110.63 (16)
C6—C1—H1A	119.5	C11—C10—C15	118.24 (19)
C3—C2—C1	118.55 (19)	C11—C10—C9	119.80 (18)
C3—C2—H2A	120.7	C15—C10—C9	121.96 (17)
C1—C2—H2A	120.7	C12—C11—C10	121.6 (2)
C4—C3—C2	121.9 (2)	C12—C11—H11A	119.2
C4—C3—Br1	119.56 (16)	C10—C11—H11A	119.2
C2—C3—Br1	118.56 (17)	C13—C12—C11	119.2 (2)
C5—C4—C3	119.1 (2)	C13—C12—H12A	120.4
C5—C4—H4A	120.5	C11—C12—H12A	120.4
C3—C4—H4A	120.5	C14—C13—C12	120.8 (2)
C4—C5—C6	120.9 (2)	C14—C13—H13A	119.6
C4—C5—H5A	119.5	C12—C13—H13A	119.6
C6—C5—H5A	119.5	C13—C14—C15	120.8 (2)
C5—C6—C1	118.64 (19)	C13—C14—H14A	119.6
C5—C6—C7	119.04 (18)	C15—C14—H14A	119.6
C1—C6—C7	122.30 (18)	O4—C15—C14	123.50 (19)
O1—C7—C6	121.99 (19)	O4—C15—C10	117.17 (17)
O1—C7—C8	120.4 (2)	C14—C15—C10	119.32 (19)
C6—C7—C8	117.61 (18)	O4—C16—H16A	109.5
O2—C8—C7	111.19 (18)	O4—C16—H16B	109.5
O2—C8—H8A	109.4	H16A—C16—H16B	109.5
C7—C8—H8A	109.4	O4—C16—H16C	109.5
O2—C8—H8B	109.4	H16A—C16—H16C	109.5
C7—C8—H8B	109.4	H16B—C16—H16C	109.5
			
C6—C1—C2—C3	0.1 (3)	C8—O2—C9—C10	171.08 (17)
C1—C2—C3—C4	0.1 (3)	O3—C9—C10—C11	170.4 (2)
C1—C2—C3—Br1	178.86 (15)	O2—C9—C10—C11	−10.3 (3)
C2—C3—C4—C5	0.1 (3)	O3—C9—C10—C15	−10.5 (3)
Br1—C3—C4—C5	−178.69 (15)	O2—C9—C10—C15	168.90 (17)
C3—C4—C5—C6	−0.4 (3)	C15—C10—C11—C12	−0.2 (3)
C4—C5—C6—C1	0.5 (3)	C9—C10—C11—C12	179.0 (2)
C4—C5—C6—C7	179.23 (18)	C10—C11—C12—C13	−0.2 (3)
C2—C1—C6—C5	−0.4 (3)	C11—C12—C13—C14	0.5 (4)
C2—C1—C6—C7	−179.03 (19)	C12—C13—C14—C15	−0.6 (4)
C5—C6—C7—O1	4.6 (3)	C16—O4—C15—C14	0.5 (3)
C1—C6—C7—O1	−176.7 (2)	C16—O4—C15—C10	−178.7 (2)
C5—C6—C7—C8	−175.25 (18)	C13—C14—C15—O4	−179.0 (2)
C1—C6—C7—C8	3.4 (3)	C13—C14—C15—C10	0.2 (3)
C9—O2—C8—C7	−76.7 (2)	C11—C10—C15—O4	179.39 (17)
O1—C7—C8—O2	4.4 (3)	C9—C10—C15—O4	0.2 (3)
C6—C7—C8—O2	−175.70 (16)	C11—C10—C15—C14	0.1 (3)
C8—O2—C9—O3	−9.5 (3)	C9—C10—C15—C14	−179.03 (18)

### Hydrogen-bond geometry (Å, °)

**Table:**

*D*—H···*A*	*D*—H	H···*A*	*D*···*A*	*D*—H···*A*
C2—H2A···O1^i^	0.93	2.45	3.360 (3)	165

Symmetry codes: (i) *x*+1, *y*, *z*.
